# What does ‘complex’ mean in palliative care? Triangulating qualitative findings from 3 settings

**DOI:** 10.1186/s12904-017-0259-z

**Published:** 2018-01-04

**Authors:** Emma Carduff, Sarah Johnston, Catherine Winstanley, Jamie Morrish, Scott A. Murray, Juliet Spiller, Anne Finucane

**Affiliations:** 1 Marie Curie Hospice, 133 Balornock Road, Glasgow, G21 3US UK; 2 School of School of Medicine, Nursing and Healthcare, University of Glasgow, 59 Oakfield Avenue, Glasgow, G12 8LL UK; 3 Faculty of Medicine, University of Edinburgh, Edinburgh, UK; 4 Faculty of Medicine, University of Aberdeen, Aberdeen, UK; 5 Primary Palliative Care Research Group, Centre for Population Health Sciences, The Usher Institute, The University of Edinburgh, Medical School, Teviot Place, Edinburgh, EH8 9AG UK; 6 Marie Curie Hospice Edinburgh, Frogston Road West, Edinburgh, EH10 7DR UK

**Keywords:** Complex need, Palliative care, Hospices, Hospital, Primary health care

## Abstract

**Background:**

Complex need for patients with a terminal illness distinguishes those who would benefit from specialist palliative care from those who could be cared for by non-specialists. However, the nature of this complexity is not well defined or understood. This study describes how health professionals, from three distinct settings in the United Kingdom, understand complex need in palliative care.

**Methods:**

Semi-structured qualitative interviews were conducted with professionals in primary care, hospital and hospice settings. Thirty-four professionals including doctors, nurses and allied health professionals were recruited in total. Data collected in each setting were thematically analysed and a workshop was convened to compare and contrast findings across settings.

**Results:**

The interaction between diverse multi-dimensional aspects of need, existing co-morbidities, intractable symptoms and complicated social and psychological issues increased perceived complexity. Poor communication between patients and their clinicians contributed to complexity. Professionals in primary and acute care described themselves as ‘generalists’ and felt they lacked confidence and skill in identifying and caring for complex patients and time for professional development in palliative care.

**Conclusions:**

Complexity in the context of palliative care can be inherent to the patient or perceived by health professionals. Lack of confidence, time constraints and bed pressures contribute to perceived complexity, but are amenable to change by training in identifying, prognosticating for, and communicating with patients approaching the end of life.

**Electronic supplementary material:**

The online version of this article (10.1186/s12904-017-0259-z) contains supplementary material, which is available to authorized users.

## Background

The presence of complex needs distinguishes terminally ill patients who would benefit from specialist palliative care (SPC) from those who could be cared for by non-specialist teams [1]. Specialist palliative care should be available for all patients with complex needs, but the nature of ‘complexity’ is not well defined [2–5]. The scope of palliative care has broadened in recent years, to include supportive care, from the point of diagnosis of any life-limiting illness to end of life [6, 7]. Simultaneously, there is a growing population of patients with chronic, long-term, multi-morbid conditions who may require SPC [4].

In addition, most people still die in hospital [8], although the preferred place of death is often at home [9–11]. Seventy-five percent of people have at least one admission to hospital in their last year of life [12]. Therefore, it is important that palliative care is seen as a core responsibility of every health care professional and that SPC services are enabled to focus their resource on patients with the most complex need in every care setting. Referrers in hospital and community are responsible for identifying complex need and explaining the need for SPC input. There is an urgent need to identify which patients would most benefit from SPC input, irrespective of diagnosis. A recent Delphi study found panelists reached consensus on 11 criteria for out-patient palliative care referral for patients with cancer. These included severe physical or emotional symptoms, request for hastened death, spiritual or existential crisis, assistance with decision making or planning, patient request for referral, delirium, spinal cord compression and brain or leptomeningeal metastases [13]. Models of complexity may be useful in determining which patients would benefit most from specialist input.

Current models highlight physical, social, psychological and spiritual health factors as contributing to patient complexity. The Cumulative Complexity model suggests that imbalances between patient workload (i.e. the impact of the responsibilities of daily life and being a patient) and patient capacity (i.e. the resources and limitations of achieving the workload) causes complexity [14]. The Vector Model of Complexity suggests that a number of factors, including socio-economic, behavioral, genetic, environmental and cultural, increase or decrease complexity depending on how they inter-relate [15]. The Complexity Framework proposed by Schaink suggests that health and social experiences, demographics, mental/physical health, and social capital all co-exist and interact within the socio-political context to cause complexity [16]. However, little is known about complexity in palliative care contexts or how appropriate these models are as illness progresses.

Murtagh et al. suggest that in high income countries, between 69 and 82% of people who die need palliative care [17]. There are a number of tools which can be used to identify people who would benefit from a palliative care approach, such as the Supportive & Palliative Care Indicators Tool (SPICT™) [18, 19], and the GSF-PIG [20], which both provide primary and secondary care clinicians with guidance. However, such tools do not help identify those with the most complex needs, who would benefit from referral to SPC, and further guidance in that regard is needed [18–21].

Exploring the factors that determine patient complexity in palliative care settings would help clinicians identify which patients are most in need of a referral for SPC. Moreover, to ensure timely, appropriate, individualized and coordinated care, professionals need a shared understanding of complex needs in the context of palliative care. This research aims to explore how professionals in three different settings understand complex needs in patients approaching the end of life, and examines areas of concordance and discrepancy.

## Methods

### Ethical approval and considerations

Ethical approval was granted from the College Ethics Review Board (CERB) at the Universities of Edinburgh and Aberdeen. Local approval was provided by the hospice and the acute teaching hospital. The Consolidated criteria for reporting qualitative research (COREQ) were used to report the study [22].

### Design

This study triangulated qualitative data from 3 different settings, within one NHS Board, by 3 researchers – primary care (JM), specialist palliative care (CW), and the acute medical unit (AMU) at a large teaching hospital (SJ).

### Settings and recruitment

No minimum sample size was defined: recruitment continued until no new themes were identified. In all settings, the participants were purposively recruited to reflect the multi-disciplinary nature of palliative care and included nurses, doctors and allied health professionals (AHPs).

### Primary Care Team (PCT)

A list of GP practices served by the local hospice was retrieved. Professionals who were potentially able to participate were also identified through clinical and academic networks and approached by their Practice Manager. Individuals interested in participating could then contact the researcher directly or indirectly (via their manager).

### Specialist Palliative Care (SPC)

The researcher (CW) was based at the local hospice in the South of the city. At the time of the study, the hospice had a total capacity of 25 inpatients. It also provides day hospice and community clinical nurse specialist services.

### Acute Medical Unit (AMU)

The AMU is situated in a large teaching hospital which serves the South of Edinburgh and accepts emergency admissions throughout Lothian. The hospital admits approximately 3600 patients per year. The AMU has 60 beds with 24 Consultant doctors. The hospital Palliative Care team, consisting of 1 Consultant physician and 2 specialist nurses is based at the hospital, meaning staff could refer directly to the team, who would then co-ordinate referral to the hospice if needed. The researcher (SJ) was on site to respond to questions and conduct interviews when staff were available.

### Data collection

Face to face, semi-structured interviews were conducted with professionals in the AMU and hospice. Due to the logistical challenges of interviewing disparate professionals in the community, telephone interviews were conducted in the primary care setting. All participants provided written consent prior to the interviews. The interviews were conducted between December 2014 and May 2015 and lasted on average 30 min. Those in the AMU and hospice were conducted in a quiet location away from the main area of work. All interviews focussed on professionals’ experiences of identifying and defining complex needs in their populations. Each researcher used a similar topic guide which covered the following core areas; role, patient population, understanding of palliative care, identification of patients who would benefit from SPC, referral processes, clinician skills, knowledge and challenges (see Additional file 1).

### Transcription and analysis

**Fig. 1:**
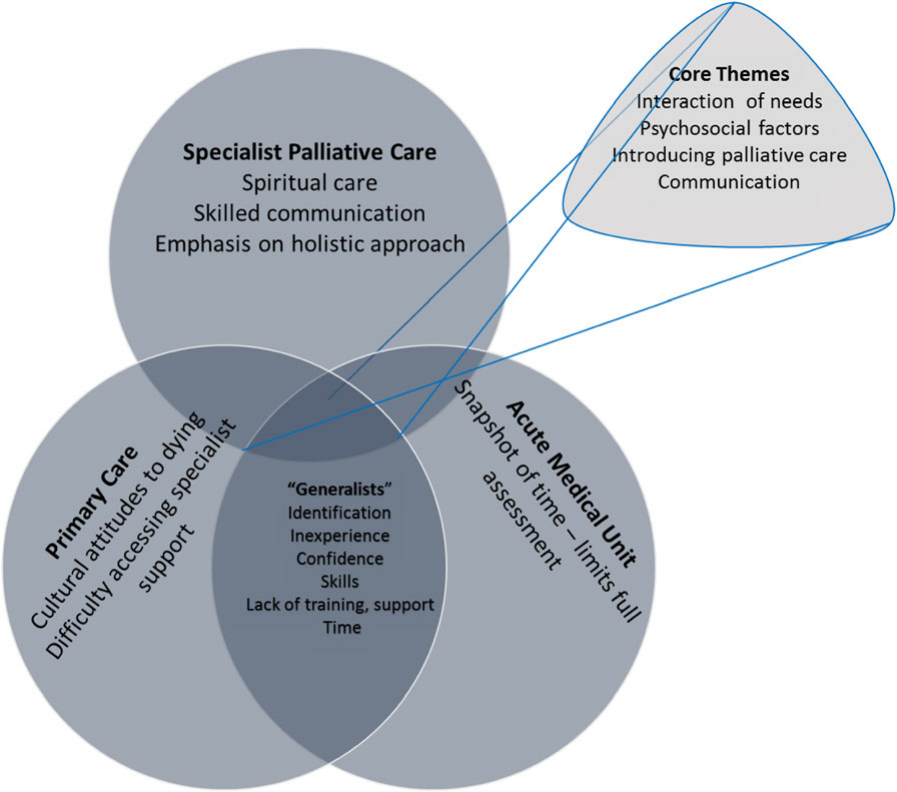
Core themes which were evident in all 3 settings and individual themes from each study

Each interview was audio-recorded, transcribed verbatim by the researchers, checked for accuracy and reread. The transcripts were coded using thematic analysis, re-read, the codes listed, revised and categorised [23-25]. Finally, themes describing the codes were developed. Following this, a workshop was convened with the research team to triangulate the findings of the three studies. The team devised a diagrammatic framework which was summarised (Fig. 1) to comprehensively extract, collate and triangulate all three sets of results [26]. Similarities and differences were summarised to develop core themes which were exclusive, or common to two or all settings. Quotes are used to illustrate the main themes.

## Results

Thirty-four interviews were conducted across the 3 settings. Table 1 illustrates the number of interviews in each setting and the multi-disciplinary nature of recruitment.

**Table 1:** Number of interviews and role of interviewees in each setting

Specialist palliative care (SPC)		Primary care team (PCT)		Acute medical unit (AMU)	
Discipline	Number	Discipline	Number	Discipline	Number
Doctors	3	GP	7	Doctors	8
Nurses	5	District/Specialist nurse	4	Nurses	5
Physiotherapist	1				
Social Worker	1				
Total	10	Total	11	Total	13
				Grand total	34

The key themes from each setting and the core themes are summarised in Fig. 1.

### Complexity arising from multiple needs

Professionals described many examples of challenging and unmet patient needs that spanned more than one dimension of palliative care – physical, psychological, social, spiritual, but it was the interaction between needs – either intrinsic (patient-specific factors) or extrinsic (factors related to the environment) that made them complex.

*“I think you realize when you, really, when you’re looking at a patient and thinking there’s this and this and this, it is getting quite complex…” (Study AMU, Doctor).*

*“...the trickiest patients are patients who’ve got a real disease, and real problems that are causing symptoms but when their symptoms are...when there is a big psychological element to their symptoms. That’s when it becomes really tricky.” (Study SPC, Doctor).*

**Psychological, social or spiritual needs interacting with the physical** was frequently highlighted.

*“I think you could have somebody who’s referred to specialist palliative care with what seems like very complex pain, nausea, physical symptoms. And then when you actually spend some time assessing them and tease things out, you realize that psychological distress is playing a large part in that and if you can treat that then actually everything else becomes a little bit easier to manage.” (Study SPC, Doctor).*

Social factors, such as difficult relationships and poor communication influenced patient complexity.

*“when their relatives are in they’re often like arguing amongst themselves, like they don’t quite know how to express...the only way they can express themselves to each other is through anger...” (Study SPC, Nurse).*

**Complexity also arose from multiple needs within a specific domain.** Patients with multiple physical symptoms, due to comorbidity or intensive therapeutic interventions, were described as complex.

*“...often these people are on complicated regimens of medication so you’re having to balance the side-effects of the medication with the benefits” (Study PCT, Doctor).*

Professionals in the AMU and PCT emphasized that a single challenging symptom, particularly uncontrolled pain or other intractable symptoms, could also create patient complexity.

*“... you’ve worked your way up the ladder and they’re on an opiate...you’ve done all of what’s very sensible, but you’ve kind of got to the end of your pain ladder and they’re still in a lot of pain.” (Study AMU, Doctor).*

Social factors also determined where a patient could be cared for. Professionals in the AMU considered patients to be complex if they could not be cared for at home.

“*Families are just exhausted, and are at breaking point and aren’t really able to support the patient anymore.” (Study AMU, Nurse).*

Specialist palliative care professionals described a holistic approach to the management of palliative patients. Spiritual needs were thought to increase perceived complexity, which was not evident in the interviews with non-specialists. For example, loss of autonomy and existential distress were common and thought to be well managed by SPC professionals.

*“And sometimes, they’re getting close to that time so, and it gets them quite...because they feel they are still here so they have a lot of existential distress about why I’m still here, what is gonna happen, I outlived my prognosis.” (Study SPC, AHP).*

This was not described by staff from the AMU.

*“I think there’s probably a bit of onus on people to reflect on their own spiritual journey and needs....We don’t really ask any questions about any of that here.” (Study AMU, Doctor).*

#### Introducing palliative care

The most appropriate time to introduce palliative care was a concern across all three healthcare settings, though professionals agreed - earlier was better. Patients with non-malignant disease were considered more complex given the uncertain disease trajectory and subsequent difficulty identifying when SPC would be appropriate.

*“I’d hope that cardiology would be talking to Palliative Care if they had a patient getting towards end stage HF and having that discussion with the patient. But if they came to the hospital, that’s difficult though because of the kind of up-down nature of the disease. It’s difficult.” (Study AMU, Doctor).*

#### Complexity arising from communication challenges

All health professionals wanted to prioritize patient preferences. However, PCT professionals described that not knowing when and how to have end of life conversations increased perceived patient complexity and management.

“*the difficulty is knowing whether it is something that they will want to talk about or not, because you don’t want to be in that situation where you force them into that conversation that they’re not wanting to have and are not ready to have...” (Study PCT, Doctor).*

Perceived complexity arose from patient’s and society’s reluctance to engage with the concept of death, dying and palliative care, which was particularly highlighted by professionals working in the community.

“*Because we’re brought up, and brought through our careers that we’re going to make people better and we can’t always make things better” (Study PCT, Nurse).*

Professionals also struggled to communicate with patients and families who did not want to discuss the future.

*“‘let’s not talk about’ and then it’ll all go away’, which actually makes it much more difficult to see that good care can be given because good care requires good communication” (Study PCT, Doctor).*

Poor communication within, and between specialties, and the multi-disciplinary team exacerbated perceived patient complexity as the quote to follow highlights – it was also a barrier to the identification and management of complex needs.

“*One aspect that is difficult is communication. With medical and nursing staff, because things can change so quickly with patients and sometimes, you know you’re halfway through an assessment, the patient’s deteriorated and maybe someone doesnae tell you, or you’re getting different information from people. So that can be a bit frustrating.” (Study PCT, AHP).*

Patients with additional communication needs, whether due to language, disability or cognitive impairment, were considered as having perceived complex need by many SPC professionals because it challenged holistic assessment, decision making and a deeper exploration of patients’ needs.

*“Increasingly these days, we’re getting patients with Alzheimer’s and dementia. I find they are quite complex in that they’re very difficult to assess properly. And their...it’s difficult to assess their needs because they can’t tell you a lot of the time how they’re feeling and I think it’s difficult to know whether you are meeting their needs or not.” (Study SPC, Nurse).*

#### Complexity arising from lack of confidence

Primary care and AMU staff identified themselves as palliative care “generalists.” Most felt they lacked confidence and the necessary skills to identify and care  for patients at the end of life and used judgment and experience as a proxy. As a result, patients were often seemingly ‘complex’ because professionals felt ill equipped in providing palliative care. Professionals valued SPC support and advice to facilitate decision making and care. Staff in AMU felt they had adequate access to SPC support, as there was a Palliative Care team based at the hospital.

*“I think I’d contact the palliative care team here to help decide when we need their help, I wouldn’t want to make that decision myself. I wouldn’t know how.” (Study AMU, Doctor).*

*“Complexities can arise for various reasons, whether that’s social, psychological, physical and I’m not sure there’s necessarily an algorithm for that. I think a lot of that comes down to judgment, really.” (Study AMU, Doctor).*

## Discussion

This exploratory study set out to understand how health professionals’ understand complex need for patients approaching the end of life. The findings suggest that health professionals across different settings identified core aspects, which have the potential to aid distinguishing those patients who would benefit most from SPC input. Some were *inherent to the patient*. For example, complexity resulted from the interaction of needs across multiple dimensions of need, or the interaction of multiple or intractable needs within one dimension of need (such as physical symptoms). The interaction between multi-dimensional aspects of wellbeing corresponds with existing research and models of complexity [13, 15, 27].

However, other factors caused *perceived complexity*, resulting from constraints of the setting, or professionals’ skills and confidence. For example, healthcare professionals’ lack of time, training, past experience and confidence in caring for patients approaching the end of life may result in perceived complexity. Staff in the AMU staff did not perceive themselves as the most appropriate people to address psychological or spiritual distress. Reluctance to manage basic psychological need at a ward-level was also found by Ewing et al. who suggested achievable, simple support measures such as information giving and reassurance were missing [28]. Furthermore, discussing inappropriate referrals as a team can present an opportunity to up-skill staff [28], improve confidence and reduce perceived complexity.

**Fig. 2:**
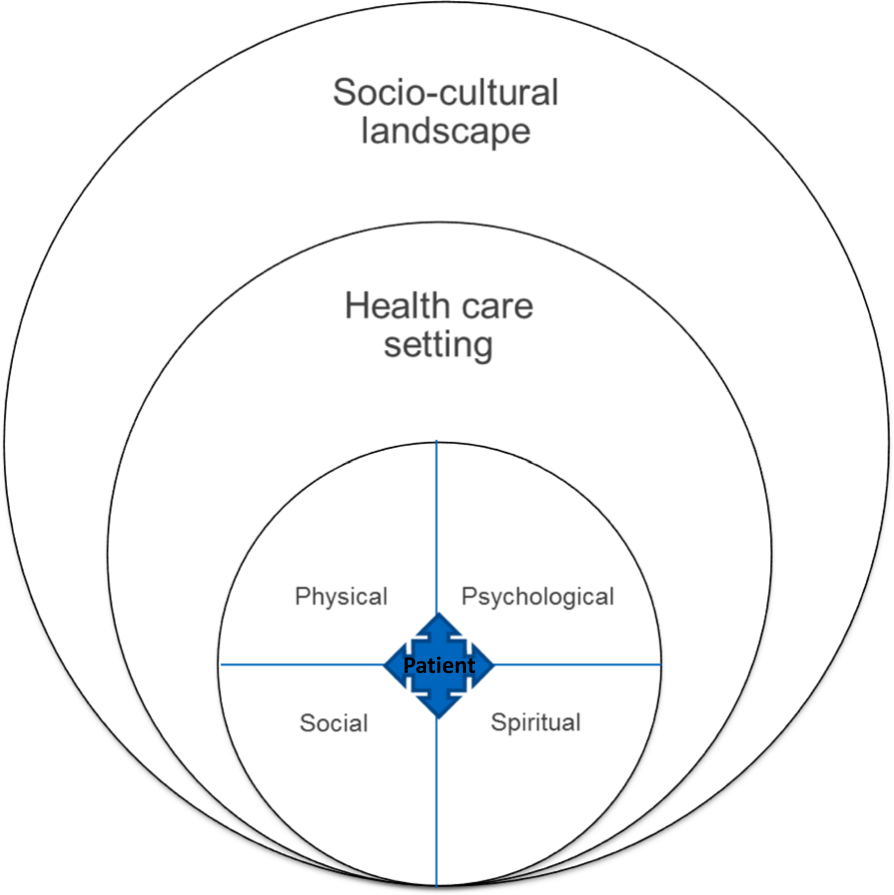
Levels of complexity

As Fig. 2 illustrates, complexity was also identified at the level of the patients, the health care environment and at the societal level. In this way, our findings relate most closely to Schaink’s Complexity Framework [16] - where health and social experiences, demographics, mental/physical health, social capital all co-exist and interact within the socio-political context to cause complexity. Professionals agreed that person-centered end of life care was paramount, but that delivery was subject to a number of contextual factors. The identification of patients who would benefit from palliative care was challenging for everyone. Patients with a diagnosis of non-malignant disease or dementia were perceived as particularly complex, given the uncertain trajectory of illness, and challenges with communication around care planning and sensitive psycho-social issues. Perceived complexity regarding dementia may be reduced when family are present and can advocate for their preferences.

### Implications

Medical and nurse training for non-specialist palliative care professionals needs to be expanded to develop skills in managing intractable symptoms, communicating with patients and their families, and identifying and addressing basic psychological and spiritual distress. We would advocate for hospice outreach education, particularly for primary care professionals who, unlike many of their colleagues in acute care, do not necessarily have access to SPC for advice. Education would also improve health professionals’ confidence, resulting in fewer patients with seemingly “complex” needs, who could be managed in the acute setting or at home, being inappropriately referred for SPC. Patients may be perceived as complex if staff feel they are unable to meet the needs of patients at the end of life and their families. Palliative care teaching during undergraduate training should be expanded for all disciplines and should include practice focused education. This should continue throughout foundation training, where practitioners of the future are able to build on their skills.

Earlier identification of patients could improve the management of complex needs. If a patient is already identified for palliative care, a more integrated approach between health and social care should be achievable. Tools such as SPICT™ [18, 19] need to be used more, to assist professionals identify patients who are likely to benefit from palliative care. Earlier identification of patients who would benefit from palliative care would also ensure that advance care plans were in place and, combined with an integrated and person-centered approach, context-related complex needs may be reducible. Successful communication should prevent inappropriate admission to hospital and improve flow of patients to the most appropriate setting.

In addition to earlier identification, it is necessary to monitor patient needs over time – particularly those with a non-malignant and fluctuating illness trajectory. For instance, psychological and spiritual distress can be very high at particular times such as diagnosis [29], when treatment ends or during acute exacerbations or relapses - the recognition that complex needs will vary over time is important and consequently, flexible interventions are required. Greater flexibility in terms of SPC input are recommended. For instance, specialists should be available to intervene early, if required, to manage complex needs and then take a step back if the same needs are no longer evident or have a more visible role in multi-disciplinary team meetings [30].

### Strengths and limitations

This paper describes complex palliative care need from three settings which is a strength. However, this was only within one NHS Board area and there were multiple interviewers. A similar topic guide was used in each and the projects overseen by a core team. The participants were recruited purposively, so may have had a particular interest in palliative care. However, recruitment was from a number of disciplines, and thus strengthening the scope and relevance of the results to inform practice and future work in a number of areas. It is noteworthy that the AMU setting had a unique relationship with the Palliative Care team in the hospital, who were on site, and decided who was suitable for specialist palliative care support. Areas with a different model and levels of specialist palliative care support may experience different issues.

## Conclusions

Palliative care services need to recognize that while complexity may be defined by inherent patient needs, in one of more dimension of care, which are difficult to manage, it is also perceived by clinicians considering referral. Perceived complexity is highly dependent on individual factors such as time constraints, training, alternative treatments and referrals, resources and relationships with specialists. Inherent patient complexity is theoretically a relatively predictable source of need for access to SPC services, although it is important to acknowledge that many complex patients, such as those with dementia and multi-morbidity are not referred to specialist palliative care. By comparison, perceived complexity is *‘in the eye of the beholder’* and may be amenable to training and support. Professionals in all settings require confidence to start palliative care through identification, early in the illness trajectory and care planning. However, education is essential to ensure health professionals in all settings feel competent and confident to provide person-centered end of life care for all. Palliative care models of the future need to be flexible and sensitive to the changing nature of complex need, yet simultaneously devise a strategy to integrate palliative care with disease management in non-specialist areas.

## Additional file

Additional file 1: Defining complexity in palliative care – Topic guide for interviews. (DOC 26 kb)

## Abbreviations

AMU: Acute medical unit
CERB: College Ethics Review Board
PHT: Primary care team
SPC: Specialist palliative care
