# Supplementary material for: What does ‘complex’ mean in palliative care? Triangulating qualitative findings from 3 settings
Source: BMC Palliat Care. 2018 Jan 4;17:12. doi: 10.1186/s12904-017-0259-z (PMC5753489; doi:10.1186/s12904-017-0259-z)
Supplement: Additional file 1: — Defining complexity in palliative care – Topic guide for interviews. (DOC 26 kb) [file 12904_2017_259_MOESM1_ESM.doc]

Defining complexity in palliative care - Interview Topic Guide

- Role in referral
- Defining palliative care
- Identifying ‘complex’ patients
- Referral process
- A recent *experience* of referral
- Challenges and improvements: Current systems achieve best results?

***Can we I start by finding out a bit more about you…***

Can you tell me a bit about your job/role?

What is your involvement in referring/ recommending referral in palliative patients?

***What people understand by the term ‘palliative’ differs…..***

What are your understandings of palliative care and specialist palliative care?

What do you think patients understand by the terms palliative care and specialist palliative care?

***Complex Needs…***

How would you define “complex needs” in relation to palliative care?

How do you identify which patients with palliative needs are most complex?

Who do you think should go to the hospice?

***Referral process***

Are there reasons you would not recommend referral of a complex patient?

What do you think is the major reason for referral to hospice (physical, psychological, social, spiritual)?

How does the patient’s diagnosis impact on your decision to refer?

It seems that the timing of referral can be difficult. How does the stage of the patient’s disease affect your decision to refer?

Thinking about limited resources, is there a criteria or guidance on who to refer? If not, what would you include in a potential criteria?

Would you be able to describe a recent experience of referring a patient?

***Challenges and improvements***

In your opinion, does what happens match up to what you think would be the best system?

In you opinion are there any ways either defining complex patients or the referral system could be improved?
